# A Scalable and Robust Personal Health Management Textile with Multiple Desired Thermal Functions and Electromagnetic Shielding

**DOI:** 10.1002/advs.202400687

**Published:** 2024-04-22

**Authors:** Litao Tang, Bin Lyu, Dangge Gao, Yingying Zhou, Yunchuan Wang, Fangxing Wang, Zhangting Jia, Yatong Fu, Ken Chen, Jianzhong Ma

**Affiliations:** ^1^ College of Bioresources Chemical and Materials Engineering Shaanxi University of Science & Technology Xi'an 710021 China; ^2^ National Demonstration Center for Experimental Light Chemistry Engineering Education Shaanxi University of Science & Technology Xi'an 710021 China; ^3^ Xi'an Key Laboratory of Green Chemicals and Functional Materials Shaanxi University of Science & Technology Xi'an 710021 China

**Keywords:** durability, electric heating, electromagnetic interference shielding, personal health management, radiative heating

## Abstract

The development of functional textiles combining conventional apparel with advanced technologies for personal health management (PHM) has garnered widespread attention. However, the current PHM textiles often achieve multifunctionality by stacking functional modules, leading to poor durability and scalability. Herein, a scalable and robust PHM textile is designed by integrating electrical, radiative, and solar heating, electromagnetic interference (EMI) shielding, and piezoresistive sensing performance onto cotton fabric. This is achieved through an uncomplicated screen‐printing process using silver paste. The conductivity of the PHM textile is ≈1.6  ×  10^4^ S m^−1^, ensuring an electric heating temperature of ≈134 °C with a low voltage of 1.7 V, as well as an EMI shielding effectiveness of ≈56 dB, and human motion monitoring performance. Surprisingly, the radiative/solar heating capability of the PHM textile surpasses that of traditional warm leather. Even after undergoing rigorous physical and chemical treatments, the PHM textile maintains terrific durability. Additionally, the PHM textile possesses maneuverable scalability and comfortable wearability. This innovative work opens up new avenues for the strategic design of PHM textiles and provides an advantageous guarantee of mass production.

## Graphical abstract

A novel Personal Health Management textile is proposed by integrating electrical heating, radiative heating, EMI shielding, and piezoresistive sensing performance onto a cotton fabric, which is achieved through an uncomplicated screen‐printing process using silver paste.

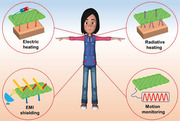

## 1 Introduction

With the intensification of climate change, the intensity and frequency of extreme cold weather are gradually increasing.^[^ ^1^, ^2^, ^3^ ^]^ Maintaining a stable body temperature is crucial for human health in frigid climates.^[^ ^4^, ^5^ ^]^ Statistics show that low temperatures account for 94.5% of fatalities caused by inclement conditions.^[^ ^6^ ^]^ To obtain sufficient warmth, humans usually adapt some traditional measures such as air‐conditioning, central heating, and stoves.^[^ ^7^ ^]^ However, these methods are energy‐intensive and wasteful, as they require a significant amount of energy to heat the surrounding space.^[^ ^8^, ^9^ ^]^ In addition, the rapid advancement in 5th generation mobile communication and the prosperity of mobile electronic devices create massive electromagnetic interference (EMI), which poses risks to human health and environment.^[^ ^10^, ^11^ ^]^ Therefore, it is crucial to develop advanced functional materials to ensure personal health management (PHM) for humans.

Functionalized wearable materials are playing an essential part in the development of PHM, providing thermal comfort for humans and protection from electromagnetic pollution.^[^ ^12^, ^13^, ^14^, ^15^ ^]^ Currently, polymer‐based film materials with PHM functions are widely reported due to their lightweight nature and the processability of conductive networks. For example, a carbon fiber@NiCo/polyimide film demonstrates efficient EMI shielding and electric heating through the use of magnetic NiCo and conductive carbon fibers.^[^ ^16^ ^]^ Another example includes a polyurethane/graphite fiber composite film decorated with an ammonium polyphosphate (APP) and transition metal carbides/nitrides (MXene), achieving EMI shielding and electric heating through the assembly of APP wrapped with MXene and graphite fiber.^[^ ^17^ ^]^ Nonetheless, the modular stacking increases the stiffness of these PHM film materials,^[^ ^18^, ^19^ ^]^ while poor permeability and mechanical mismatches with the human body of these films usually restrict the wearable comfort of humans.^[^ ^20^ ^]^

Textiles are crafted from fibers to provide the desired wearability necessary for ensuring comfort during everyday human activities.^[^ ^21^ ^]^ It is a promising alternative for PHM by endowing textiles with advanced thermal functions (e.g., active electric heating or passive radiative heating) and EMI shielding by integrating multifunctional materials into textiles. Hitherto, various methods, including vacuum filtration,^[^ ^22^ ^]^ spraying,^[^ ^14^ ^]^ and in‐situ synthesis,^[^ ^23^ ^]^ have been employed to fabricate PHM textiles. For instance, a lightweight MXene‐coated nonwoven fabric wearable heater was developed using the spraying‐drying technique,^[^ ^24^ ^]^ showcasing impressive electric heating (263 °C at 5 V) and EMI properties (35.7 dB for a single‐layer fabric). Furthermore, a smart wearable textile was engineered by inducing the in‐situ growth of AgNWs on cotton fabrics with caffeic acid,^[^ ^25^ ^]^ resulting in an EMI shielding efficiency of 38.2 dB and favorable electrical/photo‐thermal conversions. While numerous attempts have been carried out to improve PHM textiles to meet the increasing demand for personal thermal comfort and protection against electromagnetic pollution, there remains a lack of focus on the scalability and durability of PHM textiles with desired thermal functions and EMI shielding. As a result, current PHM textiles are predominantly confined to laboratory demonstration stages.

Herein, we propose an efficacious strategy for developing a PHM textile (AgPA/CF) with scalable preparation and durability by integrating active electric heating, EMI shielding, radiative/solar heating, and piezoresistive sensing into wearable textiles. The strategy involves decorating inexpensive cotton fabric with a silver paste composed of silver flakes and epoxy resin using a simple screen‐printing method. Due to outstanding conductivity, the PHM textile exhibits precise electric heating regulation, reaching a high surface temperature of ≈134 °C with just 1.7 V. Additionally, it possesses superb EMI shielding effectiveness (≈56 dB) and can detect a wide range of human motions. The strong interface adhesion of epoxy resin in the silver paste ensures that the desired conductivity of the PHM textile remains high even after rigorous physical and chemical treatments (e.g., friction, bending, washing, tearing of tape, acid and alkali soaking, and exposure to high and low temperatures), highlighting its excellent durability. Moreover, the silver paste applied to the cotton fiber surface gives the PHM textile low mid‐infrared emissivity (≈46%), ensuring the radiative heating capability. Notably, the introduction of the silver paste does not sacrifice the wearability of PHM textiles. This innovative work paves the pathway for the fabrication of scalable and durable PHM textiles with advanced functions.

## 2 Results and Discussion

### 2.1 Fabrication and Characterization

**Figure 1:**
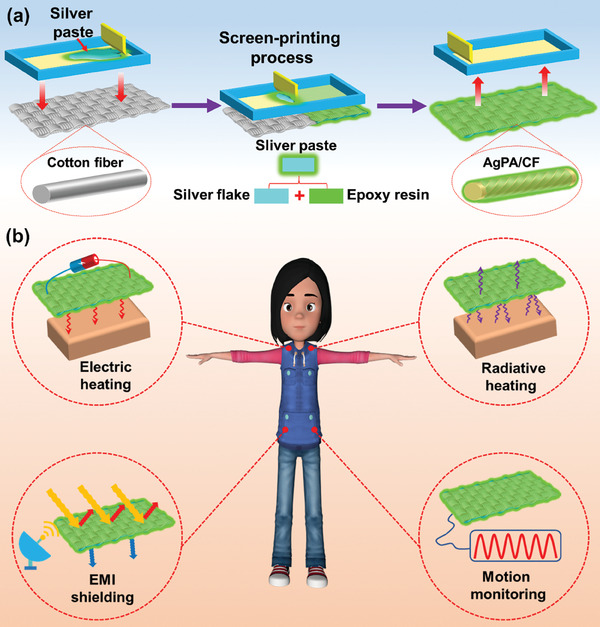
a) Schematic of the AgPA/CF preparation. b) The multifunctionality of the AgPA/CF with electric heating, radiative heating, EMI shielding, and human motion monitoring.

The PHM textile (AgPA/CF) was fabricated by applying the silver paste consisting of silver flakes and epoxy resin onto the cotton fabric using a straightforward one‐step screen‐printing process, depicted in **Figure** **1**a. The epoxy resin ensures the adhesion strength between the silver flakes in the silver paste and the cotton fibers due to its strong adhesion. The combination of cotton fabric and conductive silver paste endows the AgPA/CF with multiple desired PHM functions, including electric heating, radiative heating, EMI shielding, and human motion monitoring (Figure 1b).

**Figure 2:**
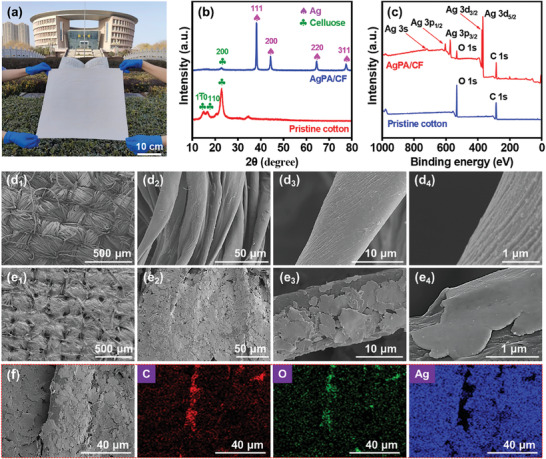
a) The digital image of the AgPA/CF at a pilot scale. b) The X‐ray diffraction pattern of the AgPA/CF and pristine cotton. c) The XPS survey spectra of the pristine cotton and AgPA/CF. d,e) The SEM images of (d) the pristine cotton and (e) AgPA/CF. f) The SEM and EDS images of the AgPA/CF and corresponding elemental mapping.

**Figure** **2**a depicts the large‐area AgPA/CF at a pilot scale by the screen‐printing technology. It can be seen that we can easily fabricate a sample of 40  ×  40 cm^2^, which is rare in other reports on PHM textiles. This indicates that the AgPA/CF possesses great potential for large‐scale production and can expand to clothing fabrics. XRD and XPS were employed to confirm the composition of the AgPA/CF surface. Figure 2b shows the XRD patterns of the pristine cotton and AgPA/CF. Compared to the pristine cotton, strong diffraction peaks at 38.3, 44.5, 64.6, and 77.6° appear in the pattern of the AgPA/CF, corresponding to the 111, 200, 220, and 311 crystal faces of elemental silver.^[^ ^26^ ^]^ As shown in Figures 2c and S1 (Supporting Information), the strong characteristic peak of Ag is detected in the XPS spectrum, and the Ag 3d peak is resolved into Ag 3d_5/2_ and Ag 3d_3/2_ of Ag^0^,^[^ ^27^ ^]^ which further demonstrates the existence of Ag. These results indicate that silver paste is introduced on the surface of the cotton fibers.

SEM images reveal the micro morphologies of the pristine cotton and AgPA/CF. As shown in Figure 2d, the surface of the pristine cotton fibers is relatively clean, and clear textures and grooves can be observed on the surface of the cotton fibers. Figure 2e depicts the micro morphologies of the AgPA/CF. It can be seen that numerous silver flakes appear on the surface of cotton fibers after screen printing. As shown in Figure 2e_4_, the surface of the silver flakes is comparatively smooth, which is beneficial for reflecting mid‐infrared (mid‐IR) radiation of the human body. From Figure S2 (Supporting Information), despite the introduction of a large number of silver flakes, the gaps on the surface of the textile still remain preserved, ensuring the positive air and water vapor permeability of the AgPA/CF. Figure 2f shows the element distribution of the AgPA/CF surface. The distribution of the Ag element from the sliver flakes is similar to C and O elements from cellulose, indicating the homogeneity of sliver flakes on the surface of cotton fibers.

### 2.2 Electric Heating Performance

**Figure 3:**
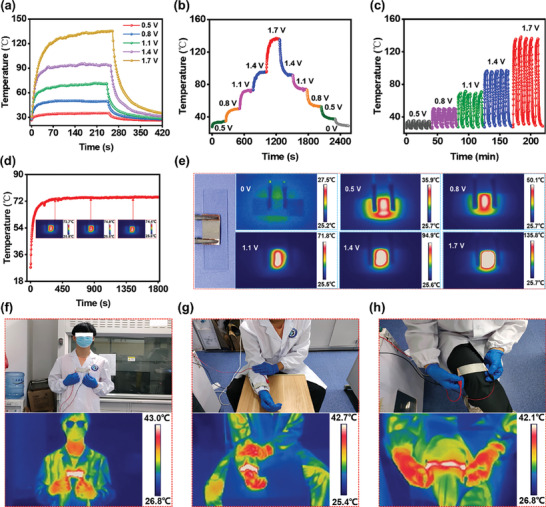
a) The real‐time temperature of the AgPA/CF under different input voltage. b) The tailored electric heating temperature of the AgPA/CF at the voltage from 0.5 to 1.7 V and from 1.7 to 0.5 V. c,d) The stability of cyclic electric heating (c) and continuous electric heating (d). e) The infrared images of the AgPA/CF at different input voltages. f–h) The optical images and corresponding IR images of the AgPA/CF covering the human chest (f), wrist (g), and knee (h).

Active thermoregulation refers to heating the microenvironment of the human body via auxiliary external energy inputs.^[^ ^28^ ^]^ Electric heating can intuitively optimize the individual thermal comfort temperature against a wider ambient temperature change by adjusting the applying voltage. As shown in Figure S3 (Supporting Information), the conductivity of the AgPA/CF is ≈1.6  ×  10^4^ S m^−1^, which endows the AgPA/CF with the desired electric heating capacity. The electric heating performance was characterized by employing a thermocouple and an infrared camera. **Figure** **3**a depicts the real‐time temperature of the AgPA/CF under different input voltages. The electric heating temperature shows a gradient‐increasing trend with the increase of voltage, which is ≈35, ≈50, ≈71, ≈94, and ≈134 °C at 0.5, 0.8, 1.1, 1.4, and 1.7 V, respectively. This phenomenon can be attributed to the fact that the temperature of electric heating is directly proportional to the input voltage, which is in accordance with Joule's law (*Q* = *U* ^2^ × *R* ^−1^ × *t*, where *Q*, *U*, *R*, and *t* are the generated heat, the input voltage, the electrical resistance, and the electric heating time, respectively^[^ ^29^ ^]^). Precise electrothermal regulation is necessary for electric heating textiles. Thus, the electrothermal response of AgPA/CF at transformed applied voltages was evaluated. As shown in Figure 3b, the electric heating temperature can be easily regulated by tailoring the input voltage either from 0.5 to 1.7 V or 1.7 to 0.5 V, indicating the dependence of electrothermal temperature on the applied voltage. Electrothermal stability is a key to ensuring the service life of electric heating textiles. As shown in Figure 3c, after 5 cycles at each voltage (0.5, 0.8, 1.1, 1.4, and 1.7 V), the electric heating temperature exhibits the high consistency corresponding to the loading and unloading of the input voltage, indicating superior cyclic electrothermal stability. Besides, within a heating time of up to 1800 s at 1.1 V, the electric heating temperature of the AgPA/CF has almost no fluctuations (Figure 3d), which suggests the terrific continuous electric heating stability.

The uniform heat distribution is regarded as a momentous criterion for electrical heaters.^[^ ^30^ ^]^ We investigated the surface heat distribution of AgPA/CF at different input voltages by an infrared camera. As shown in Figure 3e, the AgPA/CF possesses the homogenous heat distribution at the voltage of 0.5–1.7 V. Furthermore, the AgPA/CF, as an excellent flexible electric heater, exhibits excellent electrical heating effects in various parts of the human body. As shown in Figure 3f–h, the AgPA/CF can be utilized to provide thermal comfort temperature for the human chest (Figure 3f), wrist (Figure 3g), and knee (Figure 3h), suggesting the enormous application prospects of AgPA/CF in personal health management.

**Figure 4:**
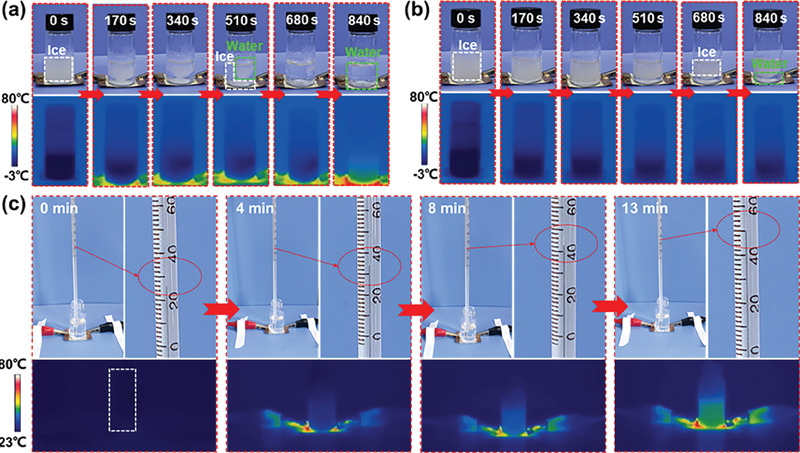
a,b) The electric heating deicing performance of AgPA/CF at 1.1 V input voltage (a) and without input voltage (b). c) The electric heating water performance of AgPA/CF at 1.1 V.

Under extreme cold conditions, ice accretion on surfaces is a universal phenomenon, which may restrict the heating performance of PHM textiles. The electric heating deicing performance of the AgPA/CF was tested, as shown in **Figure** **4**a. The ice in glass bottles can completely melt within 840 s when the input voltage of AgPA/CF at the bottom of the bottle with ice is 1.1 V. However, the ice in the bottle only melted a small part within 840 s when the input voltage is 0 V (Figure 4b). This result shows the remarkable electric heating deicing performance of the AgPA/CF. In addition, endowing electrothermal textiles with electric heating water ability can solve the short‐term need for people to drink hot water in cold conditions. Figure 4c shows the electric heating water performance of the AgPA/CF. It can be seen that the water can be heated from 26 to 50 °C within 13 min at 1.1 V, which meets the drinking demands of humans.

Therefore, the achieved electric heating textile with superior electrothermal temperature, electrothermal response, cyclic and continuous stability, uniform heat distribution, electric heating deicing, and electric heating water capability has enormous potential in PHM and practical applications.

### 2.3 Radiative/Solar Heating Performance

In cold conditions, more than 50% of stationary human body heat is dissipated in the form of infrared radiation.^[^ ^31^ ^]^ The radiative heat flux can be obtained according to the Stefan–Boltzmann law, as shown in the formula below:

(1) $$q_{\mathrm{rad}}\approx \sigma \varepsilon_{\mathrm{fab}}\left(T_{\mathrm{fab}}^{4}-T_{\mathrm{amb}}^{4}\right)$$

where *q*_rad_ is the radiative heat flux, σ is the Stefan–Boltzmann constant, *ε*_fab_ is the surface emissivity of the fabric, *T*_fab_ is the surface temperature of the fabric, and *T*_amb_ is the ambiance temperature.^[^ ^32^ ^]^ As can be seen, the textile with lower surface emissivity loses less heat than the textile with higher surface emissivity. Thus, endowing textiles with lower mid‐IR emissivity can intercept the thermal radiation dissipation of the human body, and then enhance the temperature of the human skin microenvironment.

**Figure 5:**
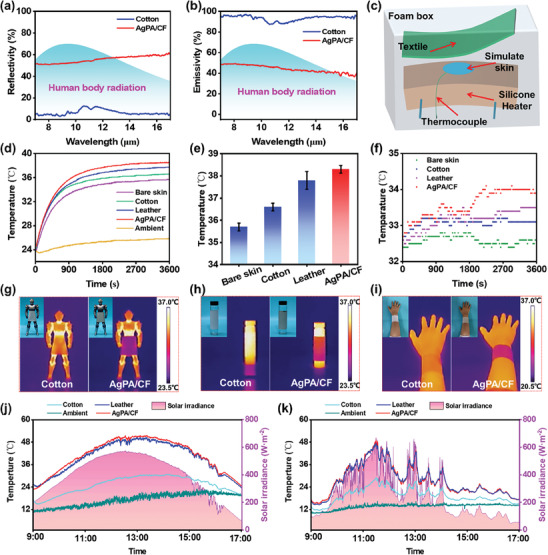
a,b) The mid‐IR reflectivity (a) and emissivity (b) of samples. c) Schematic diagram of testing device for radiative heating. d,e) Real‐time temperature curves (d) and final temperature (e) of artificial skin and artificial skin covered with samples. f) Real‐time temperature curves of human skin and human skin covered with samples. g–i) Digital and IR images of toy model (g), glass bottle with DI water (h), and human arm (i) covered with samples. j,k) Real‐time temperature curves of samples on a sunny day (j) and a cloudy day (k).

**Figures** **5**a and S4 (Supporting Information) depict the mid‐IR reflectivity and transmissivity of the pristine cotton and AgPA/CF. It can be seen that the AgPA/CF possesses higher mid‐IR reflectivity than the pristine cotton, and the mid‐IR transmissivity of the pristine cotton and AgPA/CF tends to 0. The mid‐IR emissivity is calculated according to the thermal radiation relation, i.e., *ε* = 1‐ *ρ – τ* (where *ε*, *ρ*, and *τ* represent emissivity, reflectivity, and transmissivity, respectively). In Figure 5b, the weighted average mid‐IR emissivity at 7–17 µm is calculated to be 46.2%, which is much lower than that of the pristine cotton (93.6%). The result indicates that compared to pristine cotton, the AgPA/CF possesses a stronger capability to intercept the thermal radiation of the human body.

To evaluate the radiative heating performance of the AgPA/CF, we designed a testing device consisting of textiles, a foam box, a simulated skin, a silicone heater, and a thermocouple, as shown in Figure 5c. Figure 5d presents the real‐time temperature curves of the artificial skin and artificial skin covered with cotton, leather, and AgPA/CF during the radiative heating measurement. The temperature rising rate of artificial skin covered with the AgPA/CF is the highest and the temperature tends to stabilize after 2700 s.

As shown in Figure 5e, after 3600 s, the artificial skin covered with AgPA/CF reaches the highest final temperature (38.3 °C), which is 2.6, 1.7, 0.5 °C higher than that of the artificial skin (35.7 °C), the artificial skin covered with cotton (36.6 °C), and the artificial skin covered with leather (37.8 °C), respectively. We also applied the AgPA/CF to the human body to detect its actual radiation heating performance. As shown in Figure 5f, after 45 min, the temperature of human skin covered with the AgPA/CF tends to stabilize and it is higher than that of human skin covered with leather and cotton. Notably, the thickness of the leather is ≈10 times that of the AgPA/CF. Thus, the above results fully confirm the excellent radiative heating performance of the AgPA/CF.

IR images in Figure 5g–i visually reveal the thermal radiation interception ability of the AgPA/CF. We heat the toy model and the bottle containing DI water to ≈36 °C to simulate the core temperature of the human body, as shown in Figure 5g,h. From the IR images, the parts of the AgPA/CF covering the toy model and the bottle containing DI water show a colder color, while the cotton covering parts present a warmer color. In Figure 5i, the IR images of human arms covered with the AgPA/CF and cotton display an analogous appearance similar to Figure 5g,h. This is because that the low mid‐IR emissivity of the AgPA/CF is able to obstruct the permeation of the thermal radiation generated from the human body and intercept most of the dissipation of thermal radiation. Cotton fabric has high mid‐IR emissivity and cannot block the dissipation of thermal radiation. Therefore, the IR camera can detect the thermal radiation of a human arm covered with cotton, while it can hardly catch the thermal radiation of a human arm covered with AgPA/CF. These reasons result in the different colors of the cotton fabric and AgPA/CF from the IR images.

In view of the vast abundance and omnipresence of outdoor solar irradiation, endowing PHM textiles with solar heating performance is very necessary, which can warm up the human body by capturing as much solar energy as possible in cold outdoor environments.^[^ ^33^ ^]^ As shown in Figure S5 (Supporting Information), the solar spectrum absorptivity of the AgPA/CF is 55.2%, which is higher than that of the pristine cotton (≈11%), indicating the remarkable sunlight capture ability of the AgPA/CF. This is because Ag is capable of achieving broadband solar absorption and thus possesses favorable solar heating capability.^[^ ^34^ ^]^ The measurement of the actual solar heating performance outdoors was conducted on the roof of Shaanxi University of Science and Technology (east longitude: 108^◦^58^′^; northern latitude: 34^◦^23^′^).

Figure 5j shows the real photothermal temperature curves of samples outdoors on a sunny winter day. The peak solar heating temperature of the AgPA/CF is 51.4 °C, which is ≈1 and ≈21 °C higher than that of the black leather and pristine cotton. Notably, leather is an effective fabric for keeping the human body warm, and therefore leather is widely utilized in frigid regions. Compared to black leather, the higher photothermal temperature of the AgPA/CF demonstrates the excellent outdoor thermal management capability of the AgPA/CF. The photothermal performance of the AgPA/CF on a cloudy day was also evaluated. As shown in Figure 5k, even on a cloudy day with an average temperature of 13.6 °C, the peak photothermal temperature of the AgPA/CF (49.0 °C) is 1.7 and 20 °C higher than that of the black leather (47.3 °C) and pristine cotton (29 °C), further indicating the superb solar heating performance of the AgPA/CF.

### 2.4 EMI Shielding Performance

**Figure 6:**
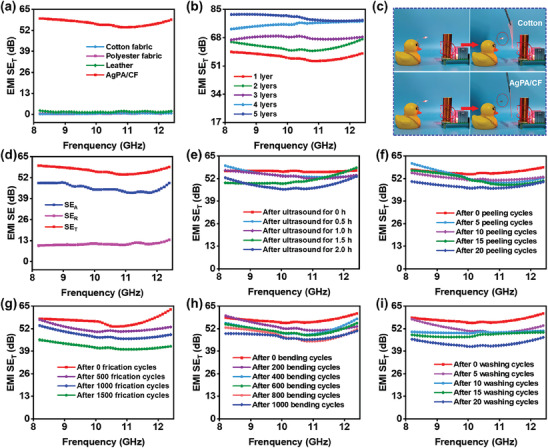
a) EMI shielding performance of different samples. b) EMI shielding performance of AgPA/CF with different thicknesses. c) The digital image of the pristine cotton and AgPA/CF shielding electromagnetic waves from Tesla coil. d) The SE_A_, SE_R_, and SE_T_ values of AgPA/CF. e–i) EMI shielding performance after ultrasound (e), peeling (f), frication (g), bending (h), and washing (i) cycles.

In the era of booming microelectronics and 5th‐generation mobile communication technology, it is of great necessity to develop flexible ultra‐efficient EMI shielding materials to promise human health and stable operation of electronic equipment.^[^ ^35^, ^36^ ^]^ The introduction of high‐conductive silver paste ensures the outstanding EMI shielding performance of the AgPA/CF. **Figure** **6**a shows the total EMI shielding effectiveness (SE_T_) value of the AgPA/CF, normal cotton, polyester fabric, and leather. Obviously, the SE_T_ value of normal cotton, polyester fabric, and leather is almost zero, while the SE_T_ value of the AgPA/CF can reach up to ≈56.4 dB, which indicates that AgPA/CF is able to shield the vast majority of X‐band electromagnetic waves. Moreover, as shown in Figure 6b, when the thickness increases to ≈1300 µm by stacking four layers of AgPA/CF, the EMI shielding effectiveness is ≈76 dB, which suggests that the EMI shielding effectiveness is greatly influenced by the thickness of the textile. However, the EMI shield effectiveness of 5‐layer AgPA/CF only increased by 4 dB compared to 4‐layer AgPA/CF. The reason is that the pores during fabric stacking can cause partial electromagnetic wave leakage.^[^ ^37^ ^]^ Figure 6c intuitively displays the EMI shielding of the AgPA/CF. It can be seen that the LED is light when the cotton fabric is placed in the electric field generated by the Tesla coil. However, the LED is unlit when the AgPA/CF is close to the Tesla coil, indicating the excellent EMI shielding performance of the AgPA/CF.

To further illustrate the EMI shielding mechanism of the AgPA/CF. The SE_T_, shielding effectiveness of the reflection (SE_R_), and shielding effectiveness of the absorption (SE_A_) are depicted in Figure 6d. It can be seen that the SE_A_ and SE_R_ are 45.6 dB and 10.8 dB, respectively. The percentage of SE_A_ in SE_T_ is ≈81%, which suggests that microwave absorption may play a dominant role during the EMI shielding process and can effectively avoid secondary electromagnetic pollution caused by electromagnetic reflection.^[^ ^38^ ^]^ The above phenomenon can be attributed to the porous structure and high conductivity of the AgPA/CF offer multitudinous interfaces for the multiple reflection, scattering, and absorption of incident waves.^[^ ^39^ ^]^

In practical application, the complex external environment places higher demands on flexible electromagnetic shielding materials. Thus, some rigorous experiments were designed to evaluate the stability of EMI shielding performance for AgPA/CF. Figure 6e plots the EMI shielding performance of the AgPA/CF after ultrasound. After ultrasound in water for 2 h, the EMI shielding effectiveness decreases from 56.2 to 48.3 dB. We also investigated the EMI shielding effectiveness stability by tearing of tape. As shown in Figure 6f, after 20 rapid tape stripping cycles, the EMI shielding effectiveness of AgPA/CF can still reach up to 47.8 dB, indicating the mechanical robustness of silver flakes on the surface of cotton fibers. Wearable fabrics can hardly avoid friction during the use process. Figure 6g describes the friction resistance of the AgPA/CF. After 1000 friction cycles, the EMI shielding effectiveness is 48.3 dB. Even after up to 1500 frictions, the AgPA/CF still possesses ≈42 dB of EMI shielding effectiveness. Similarly, after 1000 repeated bending cycles, the EMI shielding effectiveness of the AgPA/CF only decreases by 15.8% (Figure 6h). To assess the washing stability, we conducted 20 simulated washing cycles on the AgPA/CF fabric, which is equivalent to 100 home washes. In Figure 6i, as the number of washes increases, the EMI shielding effectiveness slightly decreases. After 20 washing cycles, the EMI SE value remains an EMI shielding effectiveness of 43.4 dB.

The terrific EMI shileding effectiveness and superior stability endow the AgPA/CF with more responsible application scenarios and huge potential to protect the human body from electromagnetic pollution.

### 2.5 Human Motion Monitoring Performance

Another attention of the AgPA/CF goes to its promising potential as a flexible sensor for human motion monitoring. The conductive network based on the silver paste and cotton fibers endows the AgPA/CF with the sensing performance.^[^ ^40^ ^]^ The sensing sensitivity of the AgPA/CF sensor was measured. The sensitivity (*S*) is defined as S = (Δ*I*/*I*_0_)/ΔP, where Δ*I* is the current variation under pressure, *I*_0_ is the initial current, and Δ*P* is the applied pressure. As shown in Figure S6a (Supporting Information), the Δ*I*/*I*_0_ value of the AgPA/CF sensor increases as the enhancement of the applied pressure, which implies the AgPA/CF sensor has outstanding and steady sensing feedback. The sensitivity of the AgPA/CF sensor is 2.16 and 1.13 kPa^−1^ in the low (3.2–12.8 kPa) and high (12.8–80.0 kPa) pressure range, respectively. Figure S6b (Supporting Information) shows the comparison of the sensing sensitivity of the AgPA/CF sensor with those reported in previous works.^[^ ^41^, ^42^, ^43^, ^44^, ^45^, ^46^, ^47^ ^]^ It can be seen that the sensitivity of the AgPA/CF is relatively high and superior to most of the sensors reported in previous studies.

**Figure 7:**
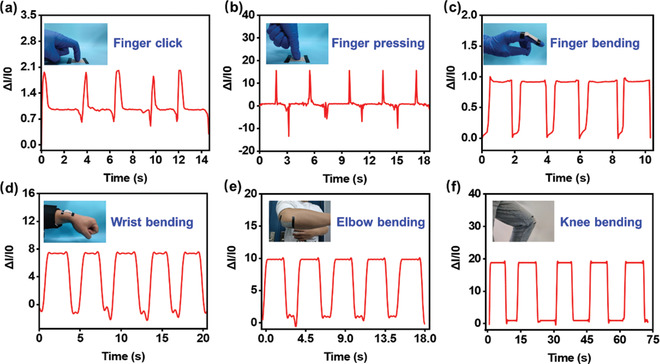
a–c) The relative current changes of the AgPA/CF during finger click (a), pressing (b), and bending (c). d–f) The relative current changes of the AgPA/CF during wrist (d), elbow (e), and knee (f) bending.

To evaluate the human motion monitoring performance of the AgPA/CF, a series of measurements were conducted. The corresponding Δ*I*/*I*_0_‐time curves are presented in **Figure** **7**. Figure 7a plots the current variation of human index finger click. When the click and click‐release actions are completed, the relative current undergoes a certain change and returns to the initial value. Similarly, the relative current also alters regularly during the pressing and pressing‐release actions, as shown in Figure 7b. This can be attributed to the fact that the conductive network of the AgPA/CF sensor is altered by external forces, which cause the electrical signal changes and present corresponding waveforms in the monitoring equipment.^[^ ^29^ ^]^

Additionally, the AgPA/CF‐based sensor can be employed to differentiate the movement of human joints. In Figure 7c, the motion signals of the forefinger are clearly identified. Especially, with the forefinger bent at 90° and unbent at 180°, the shapes of relative current signals are completely different, which indicates that the device possesses superior motion recognition performance. This is because the force generated by the finger induces the increase of charge conduction paths and the integrity of the conductive network during the process of gradual bending and reduces the resistance, leading to the increase of current.^[^ ^40^, ^41^ ^]^ In the process of the finger unbending, the charge conduction paths of the AgPA/CF‐based sensor are reduced and thus the current reduces to the initial state. Figure 7d clearly describes the current changes caused by wrist bending and unbending. When the sensor is attached to the wrist, the shape of the current signal presents regular changes, as the wrist bends and straightens. This phenomenon provides the potential to obtain movement information on human injured hands. Fortunately, the sensor also can be adapted to recognize the large amplitude movement of human joints (e.g., elbow, and knee). Figure 7e,f shows the current variation generated by the movement of the elbow and knee. Similar to the wrist movements, as the elbow and knee bend and straighten, the current signals reveal periodic variation, which also possesses desired cyclic stability. The results prove that the sensor is able to detect the movement of human joints during events such as running or mountaineering.

The above analysis demonstrates that the AgPA/CF exhibits significant advantages in terms of human motion monitoring, which possesses huge potential in signal feedback and healthcare monitoring applications in smart wearable electronics.

### 2.6 Durability

**Figure 8:**
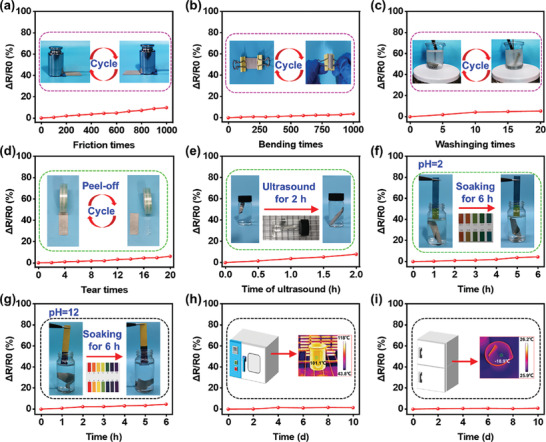
a–d) The Δ*R*/*R*_0_ changes of the AgPA/CF after multiple frictions (a), bending (b), washing (c), and tearing of tape (d). e–i) The Δ*R*/*R*_0_ changes of the AgPA/CF under long‐term ultrasound (e), soaking in acidic (f) and alkaline (g) solutions, and high (h) and low (i) temperature treatments.

It is extremely urgent to improve the durability of flexible wearable PHM textiles. Desired conductivity plays an important role in electric heating, EMI SE effectiveness, and sense. The epoxy resin in the silver paste can strongly enhance the overall mechanical stability between the silver flakes and cotton fibers by providing interfacial adhesion.^[^ ^48^ ^]^ Accordingly, the relative resistance (Δ*R*/*R*_0_) change after strict treatment can reveal the durability of flexible wearable textiles. **Figure** **8**a shows the relative resistance variability of the AgPA/CF during friction cycles. The Δ*R*/*R*_0_ gradually increases with the increase of friction times, where the Δ*R*/*R*_0_ exhibits a small increase (lower than 10%) within 1000 friction cycles. The AgPA/CF still has the desired conductivity after thousands of frictional tests due to the minor initial electrical resistance. In addition, as shown in the SEM images in Figure S7a_1‐2_ (Supporting Information), even after 1000 frictions, the silver paste still exists on the surface of the cotton fibers and does not significantly peel off, which results in no significant decrease in the conductivity of AgPA/CF after multiple frictions.

Bending is inevitable in the practical application of clothing and thus the ΔR/R_0_ of the AgPA/CF after multiple bends was measured. As described in Figure 8b, after 1000 times of bending, the Δ*R*/*R*_0_ change of the AgPA/CF is weak, indicating the superb electrical stability of the AgPA/CF. Washing stability is also an important evaluation index for PHM textiles. After 20 times of simulated washing (equivalent to 100 times of home washing), the Δ*R*/*R*_0_ only increases by 5.4%, showing the superior washing durability of the AgPA/CF (Figure 8c). Figure S7b_1‐2_ and c_1‐2_ (Supporting Information) show the SEM images of the AgPA/CF after 1000 times of bending and 20 times of simulated washing. It can be seen that silver paste is almost unaffected and still adheres to the surface of cotton fibers after multiple bending and washing, indicating the high adhesion fastness between silver paste and cotton fibers.

Tearing of tape and ultrasonic are harsh measurements for PHM textiles. Fortunately, the Δ*R*/*R*_0_ of the AgPA/CF merely increases by 6.1% (Figure 8d) and 7.8% (Figure 8e), respectively, after 20 times of fast tape tearing and 6 h of ultrasound, demonstrating the structural robustness of the AgPA/CF. Notably, the superb interaction stability between the silver flakes and the cotton fibers can also promise the durable radiative heating capability of the AgPA/CF.^[^ ^49^ ^]^

Figure 8f,g depicts the ΔR/R_0_ variability of the AgPA/CF after immersion in acidic and alkaline solutions. It can be observed that the Δ*R*/*R*_0_ exhibits an increase of less than 5% after 6 h of acid and alkaline solution soaking. As shown in Figure 8h,i, the Δ*R*/*R*_0_ is almost stable after 10 days of high (100 °C) and low (−18 °C) temperature treatment. These phenomena demonstrate that the AgPA/CF possesses excellent chemical stability.

Overall, the aforementioned results suggest that the AgPA/CF has excellent durability after a series of rigorous tests, which ensures expansive prospects in practical wearable applications.

### 2.7 Wearability

**Figure 9:**
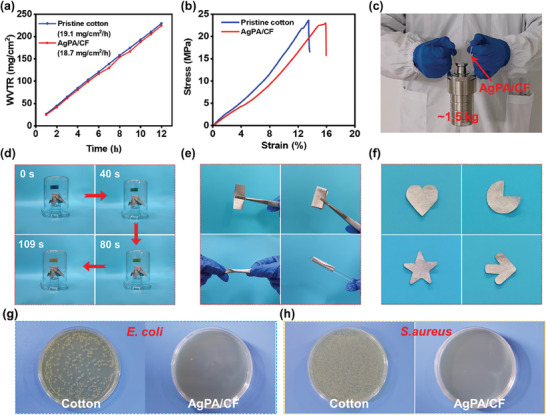
a,b) The WVTR (a) and mechanical property (b) of samples. (c) The digital image of the AgPA/CF supporting a weight of ≈1.5 kg. d–f) The air permeability (d) and flexibility (e), and tailorability (f) of the AgPA/CF. g,h) The antibacterial properties of the AgPA/CF against *Escherichia coli* (*E. coli*) (g) and *Staphylococcus aureus* (*S. aureus*) (h).

The outstanding wearability (e.g., mechanical property, water vapor permeability, air permeability, flexibility) is indispensable for PHM textiles. The wearability of the AgPA/CF was evaluated by a series of experiments. As shown in **Figure** **9**a, the water vapor transmission rate (WVTR) of the pristine cotton and AgPA/CF is 19.1 and 18.7 mg cm^−2^ h^−1^, respectively. Figure 9b shows the mechanical properties of AgPA/CF and pristine cotton. The breaking strength of the AgPA/CF is 23.0 MPa, which is slightly lower than that of the pristine cotton (23.5 MPa). The elongation at break of AgPA/CF (15.9%) is higher than that of pristine cotton (13.4%). Besides, as shown in Figure 9c, AgPA/CF can easily support a weight of 1.5 kg, indicating the desired mechanical property of the AgPA/CF.

The air permeability evaluation of the AgPA/CF was conducted according to the feature of pH test paper discoloration when exposed to acid. In brief, the transparent glass bottle containing concentrated hydrochloric acid was sealed with the AgPA/CF, and then the glass bottle was placed inside an inverted beaker with moist pH paper. As a comparison, the pristine cotton was also subjected to the same operation. In Figure 9d, the pH test paper on the top of the glass bottle sealed with the AgPA/CF changed from blue to yellow after 109 s. Notably, the time when the test paper on the top of the glass bottle sealed with pristine cotton completely turns yellow is 103 s, as shown in Figure S8 (Supporting Information). Besides, Figure 9e,f demonstrates the superb flexibility and tailorability of the AgPA/CF. It can be seen that the WVTR, mechanical strength, and air permeability of the AgPA/CF are similar to those of the pristine cotton, which ensures the potential of expanding the AgPA/CF into wearable clothing.

Cotton textiles are one of the primary mediums for transmitting viruses and bacteria in daily life,^[^ ^50^ ^]^ and thus it is of great necessity to endow AgPA/CF with exceptional antibacterial activity. The antibacterial properties of the AgPA/CF were assessed in the growth tests of *Escherichia coli* (*E. coli*.) and *Staphylococcus aureus* (*S. aureus*). As we can observe in Figure 9g,h, there is almost no *E. coli* and *S. aureus* on AgPA/CF culture dishes, while multitudinous bacterial colonies appear in the culture dishes of the pristine cotton, indicating the outstanding antibacterial activity of the AgPA/CF. This is because the Ag ions released from the AgPA/CF can attack bacterial cell membranes and cause bacterial death.^[^ ^51^ ^]^

From the aforementioned results, the desired WVTR, mechanical property, air permeability, flexibility, tailorability, and antibacterial activity of the AgPA/CF ensure its wearability, which gives the AgPA/CF great potential for practical applications.

## 3 Conclusion

In summary, a scalable and robust AgPA/CF was developed by decorating high‐conductive silver paste onto cotton fibers using a simple screen‐printing technique. The AgPA/CF exhibits superb electric heating, EMI shielding, and piezoresistive sensing capabilities. With low mid‐IR emissivity and high solar spectrum absorptivity, the AgPA/CF shows effective radiative/solar heating properties. Despite undergoing rigorous physical and chemical treatments, the AgPA/CF maintains the desired conductivity, ensuring the durability of its electric heating, EMI shielding, and sensing functions. This is attributed to the epoxy resin in silver paste enhancing the adhesion of silver flakes on the cotton fibers. Moreover, the manufacturing process of the AgPA/CF demonstrates scalability using commercially abundant raw materials. Wearability tests of the AgPA/CF yield positive results. The innovative design opens new possibilities for personal health management, potentially advancing the commercialization of health management textiles.

## 4 Experimental Section

### Materials

The cotton fabric was obtained by cutting the experimental clothing. Silver paste (01L‐7411, composition of silver flakes and epoxy resin) was purchased from Taobao (China). Sodium hydroxide (NaOH, ≥98%) was bought from Tianjin Damao Co., Ltd. Hydrochloric Acid (HCl, 36%) was provided by Sinopharm Chemical Reagents Co., Ltd. Screen mask was purchased from Taobao (China). All the chemicals were purchased as received without further purification.

### Preparation of Silver Paste/Cotton Fabric (AgPA/CF)

The cotton fabric was first cleaned by 10 g L^−1^ NaOH solution at 60 °C for 1 h. After cleaning, the cotton fabric was removed from the NaOH solution and washed several times with DI water, followed by drying. The desired viscosity of the silver paste was adjusted using a diluent. A screen made of nylon wire (mesh density 300) was used to print the conductive silver paste over the surface of cotton fabrics. During the screen‐printing process, the screen was placed above the cotton fabric and then coated with silver paste at a constant speed using a scraper. The resultant fabric was placed at 90 °C for 30 min in a vacuum oven to cure the epoxy resin in the silver paste, which increased the adhesion strength between the silver flakes and cotton fibers. After curing, the obtained composite fabric was named AgPA/CF. Any large‐sized sample can be fabricated by the screen‐printing process.

### Characterization

The microstructure and morphology of samples were obtained using a Field emission scanning electron microscope equipped with EDS (SEM, FEI‐Verios‐460, USA). The crystal structure was measured by X‐ray diffraction analysis (XRD, D8 Advance, Bruker, Germany). The chemical compositions of samples were observed via utilizing X‐ray photoelectron spectroscopy (XPS, ESCALAB 250Xi, ThermoFisher, USA). The conductivity was tested by employing a four‐probe test method (ST2253, China). The solar spectrum reflectivity and transmittance were obtained by a UV–vis–NIR spectrometer (Carry 5000, Agilent, USA) equipped with an integrating sphere. The mid‐infrared (IR) reflectivity and transmittance were measured by using an FTIR spectrometer (Nicolet IS50, ThermoFisher, USA) equipped with an infrared integrating sphere.

### Heating Performance

All experiments were carried out in accordance with all local laws and no formal approval for the experiments related to wearable technologies involving human volunteers was required by the authors’ research institution. Volunteers participated following informed consent. During the electric heating measurement, a K‐type thermocouple (JK808, China) was used to record the surface temperature of samples, and the input voltage was provided by a DC power (MS‐3010DS, China). The measurement of radiative heating performance was conducted indoors. A piece of black insulating tape was employed as the simulated skin. A silicon rubber heater was used to maintain the temperature of the simulated skin ≈36 °C by the DC power. All IR images were taken by an IR camera (DS‐2TPH16‐6AVF/W, China).

### Electromagnetic Interference Shielding Effectiveness (EMI SE)

The EMI SE test was performed by a waveguide specimen holder with a vector network analyzer (N5230A, Agilent, USA) within the frequency range of 8.2–12.4 GHz (X‐band). The calibration of the network analyzer was completed before each measurement. The power coefficients of reflection (*R*), transmission (*T*), and absorption (*A*) were calculated by measuring the scattering parameters of the reflection coefficient data S11 and the transmission data S21. The total shielding effectiveness (SE_T_), absorption effectiveness (SE_A_), reflection effectiveness (SE_R_), and multiple internal reflections (SE_M_) (SE_M_ can be negligible when SET is greater than 10 dB) were calculated as the following equations:

(2) $$R={\left|S_{11}\right|}^{2}$$

(3) $$T={\left|S_{21}\right|}^{2}$$

(4) $$\;SE_{R}=\;-10log\left(1-R\right)\;$$

(5) $$\;SE_{A}=\;-10log\left(T/\left(1-R\right)\right)\;$$

(6) $$\;SE_{T}=SE_{R}\;+SE_{A}+\;SE_{M}\;$$

### Wearability

The water vapor transmission rate of samples was evaluated by recording the mass loss of the transparent glass bottle with deionized water. After samples were used to seal the glass bottles, the glass bottles were placed in an airtight oven with a temperature of 38 ± 0.5 °C for 12 h. The data of the mass loss were recorded each hour. The mechanical properties of samples were tested by a constant‐speed tensile machine. *S. aureus* and *E. coli* were utilized as representative model bacteria. In brief, the samples (1 cm  ×  1 cm) were placed in 10 mL bacterial suspensions and subsequently shaken for 18 h at 37 °C. Following that, 0.1 mL of the treated bacterial suspension was transferred to a sterile petri dish with a 20 mL plate count agar medium via the pipette gun. After incubating at a constant temperature of 37 °C for 24 h, the antibacterial performance was evaluated by colony counting method.

## Conflict of Interest

The authors declare no conflict of interest.

## Supporting information

Supporting Information

## Data Availability

The data that support the findings of this study are available from the corresponding author upon reasonable request.
