# Supplementary material for: A Scalable and Robust Personal Health Management Textile with Multiple Desired Thermal Functions and Electromagnetic Shielding
Source: Adv Sci (Weinh). 2024 Apr 22;11(21):2400687. doi: 10.1002/advs.202400687 (PMC11151079; doi:10.1002/advs.202400687)
Supplement: Supplementary file 1 — Supporting Information [file ADVS-11-2400687-s001.pdf]

ADVANCED SCIENCE
Open Access

Supporting Information

for *Adv. Sci.*, DOI 10.1002/advs.202400687

A Scalable and Robust Personal Health Management Textile with Multiple Desired Thermal Functions and Electromagnetic Shielding

Litao Tang, Bin Lyu, Dangge Gao*, Yingying Zhou, Yunchuan Wang, Fangxing Wang, Zhangting Jia, Yatong Fu, Ken Chen and Jianzhong Ma**

A Scalable and Robust Personal Health Management Textile with Multiple Desired Thermal Functions and Electromagnetic Shielding

Litao Tang[a, b, c], Bin Lyu[a, b, c*], Dangge Gao[a, b, c*], Yingying Zhou[a, b, c], Yunchuan Wang[a, b, c], Fangxing Wang[a, b, c], Zhangting Jia[a, b, c], Yatong Fu[a, b, c], Ken Chen[a, b, c], Jianzhong Ma[a, b, c*]

a College of Bioresources Chemical and Materials Engineering, Shaanxi University of Science & Technology, Xi'an 710021, China.

b National Demonstration Center for Experimental Light Chemistry Engineering Education (Shaanxi University of Science & Technology), Xi'an 710021, China.

c Xi'an Key Laboratory of Green Chemicals and Functional Materials (Shaanxi University of Science & Technology), Xi'an 710021, China.

Corresponding Author

*E-mail address:

xianyanglvbin@163.com (B.L.);

dangge2000@126.com (D.G.);

majz@sust.edu.cn (J.M.).

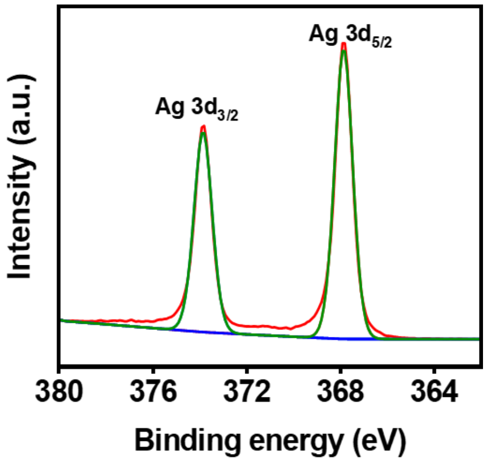

Figure S1 The high resolution Ag 3d spectrum of the AgPA/CF textile.

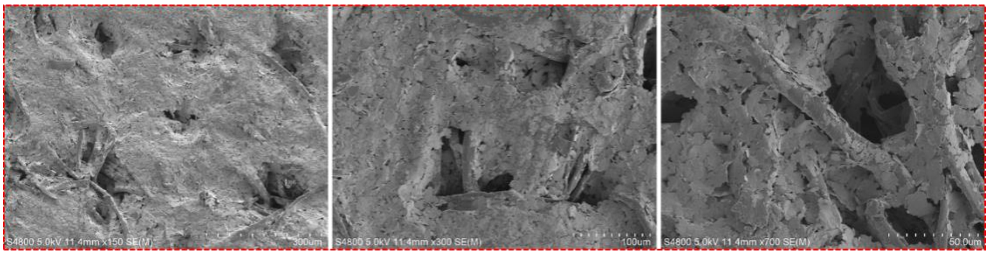
Figure S2 The SEM images of the AgPA/CF textile

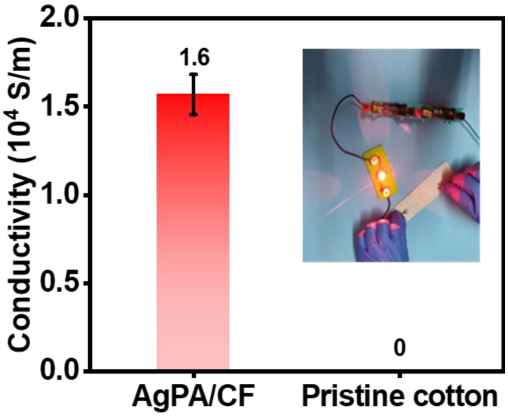

Figure S3 The conductivity of the AgPA/CF textile and pristine cotton.

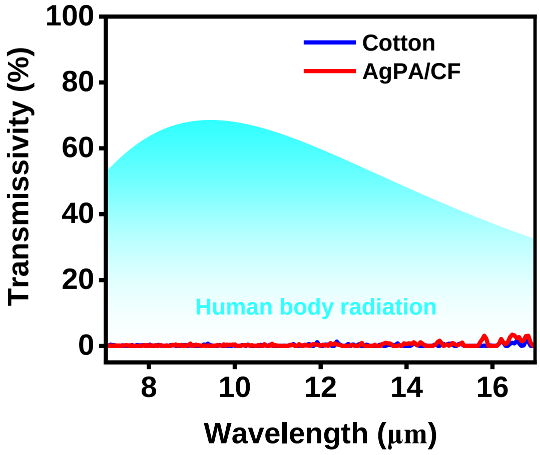

Figure S4 The mid-IR transmissivity of samples

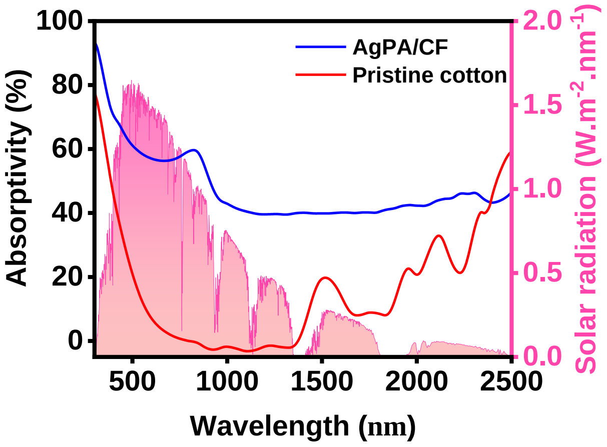

Figure S5 The solar spectrum absorptivity of the AgPA/CF and pristine cotton.

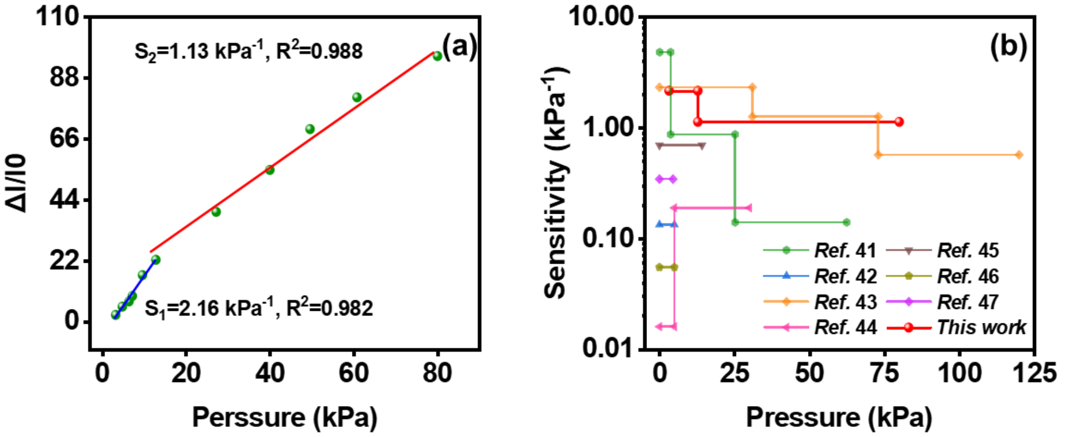

Figure S6 (a) The relative current changes of the AgPA/CF under various loaded pressure; (b) The comparison for the sensing performance of the AgPA/CF with those reported in previous works.

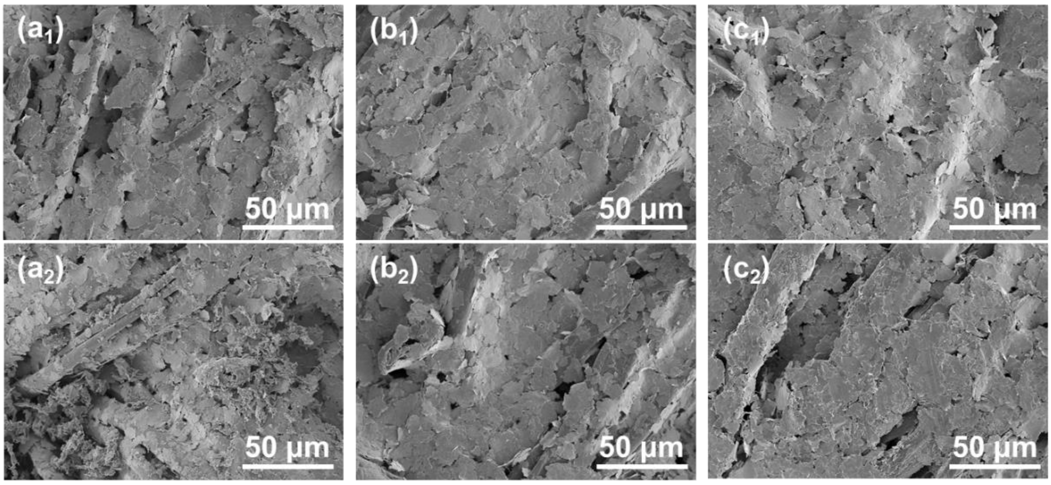

Figure S7 (a-c) The SEM images of the pristine AgPA/CF (a_1-c_1), the AgPA/CF after 1000 frication cycles (a_2), 1000 times of bending (b_2), and 20 times of simulated washing (c_2).

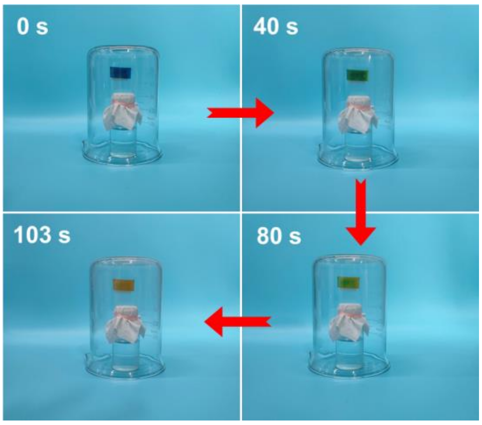

Figure S8 The air permeability of cotton.
